# Expression of collagen type 1 alpha 1 indicates lymph node metastasis and poor outcomes in squamous cell carcinomas of the lung

**DOI:** 10.7717/peerj.10089

**Published:** 2020-09-29

**Authors:** Siyuan Dong, Peiyao Zhu, Shuguang Zhang

**Affiliations:** Department of Thoracic Surgery, The first hospital of China Medical University, Shenyang, Liaoning, China

**Keywords:** Lung Squamous Cell Carcinoma, COL1A1, Lymph Node Metastasis, Biomarker, Tumor Microenvironment

## Abstract

**Background:**

Squamous cell carcinomas of the lung are an extremely common and deadly form of non-small cell lung cancers. Clinical management of the disease is dependent on staging and metastatic status. Metastasis to the lymph node is especially crucial to diagnose as it occurs at an earlier stage. However, lymphadenectomies are invasive and tumor cells may be overlooked during evaluation.There are limited approved biomarkers for predicting lymph node metastasis with squamous cell carcinomas of the lung (LSCC).

**Methods:**

Genome data of 60 tumor-adjacent samples were downloaded from Genome Expression Omnibus. We identified over-expressed HUB genes using Cytoscape as key prognostic markers. The selected markers were further evaluated based on gene ontology and overall expression levels compared to normal tissue using The Cancer Genome Atlas. We further validated these results using clinical biopsy tissue taken from squamous cell carcinoma patients.

**Results:**

Analysis of the genome expression data resulted in 13 relevant hub genes that were differentially expressed in cancerous samples. All of these genes are associated with collagen biosynthesis within the tumor microenvironment. We chose Collagen Type 1 Alpha 1 (COL1A1) as the most relevant prognostic marker due to its high number of pathway connections and over expression in the tumor microenvironment compared to the other 12 genes. Additionally, based on analysis of The Cancer Genome Atlas, tumors with higher levels of COL1A1 expression are associated with poorer overall survival. Finally, evaluation of clinical biopsy samples suggests that overexpression of COL1A1 in the LSCC microenvironment highly correlates with lymph node metastasis. These results suggest COL1A1 is a clinically relevant marker that should be used to justify lymphadenectomies.

## Introduction

Lung squamous cell carcinomas (LSCC) are a highly prevalent and lethal form of non-small-cell lung carcinoma (NSCLC). LSCC make up 20%–30% of all lung cancer cases (Dela Cruz, Tanoue & Matthay, 2011; Berardi et al., 2015) and are associated with a median 5-year survival rate of 17.6% (Lu et al., 2019). There are several factors that go into the high mortality rate, however a primary cause is that the disease is often not detected until late in the disease course (Xing et al., 2010) when symptoms begin to appear (Postmus et al., 2017). Early, asymptomatic (e.g., Stage I) NSCLC are often curable, with a median survival rate of up to 80% but outcomes worsen to <15% for later cases (Xing et al., 2010). Metastasis is often the root cause of mortality (Dillekås, Rogers & Straume, 2019), which occurs later in LSCC; therefore, early identification and accurate staging of tumors can be extremely helpful in optimizing treatment and improving patient outcomes (Collins et al., 2007).

Metastasis to the lymph nodes is often a precursor to distal metastasis (Quint, 2007). In NSCLC patients, progression to lymph nodes results in a substantial decrease in survival, and therefore is an important prognostic factor (Mountain, 2002). Lymph node sampling (lymphadenectomy) is often used to evaluate lymph node metastasis (Ettinger et al., 2014). However, several studies have shown that nodule biopsies are inadequate (Varlotto et al., 2009; Verhagen et al., 2012) and micrometasteses may be missed in routine histological evaluation (Tufman & Huber, 2010). Furthermore, unnecessary pulmonary lymphadenectomies may result in additional risks to the patient and increase the duration of in-patient care (Allen et al., 2006).

Biomarkers which can predict or identify lymph metastasis will be helpful in supplementing traditional histological evaluations and choosing effective treatments. Thus far, much of the research in this area has resulted in cellular and serum markers (Borgia et al., 2009). However, extracellular influences such as those in the tumor microenvironment are also known to play a crucial role. The proteins, immune cells, vessels, and mural cells can become dysregulated during the progression of LSCC. Identification of these markers may provide value as prognostic tools (Fang & Zhang, 2017) and druggable targets (Finan et al., 2017). For example, Meng et al. (2019) has identified differences in the immune microenvironment between LSCC and non-squamous NSCLCs and demonstrated the influence on patient prognosis. Similar work by Seo et al. (2018) identified immune subtypes that differ in LSCC and adenocarcinoma enabling more accurate diagnosis and identification. While this work is potentially useful as prognostic assessments and in maximizing the efficacy of checkpoint inhibitor therapies, it would not be useful in staging the disease (i.e., presence of lymph metastasis). Furthermore, to date, no study has evaluated proteins in the LSCC microenvironment as potential markers of lymph node metastasis.

**Figure 1:**
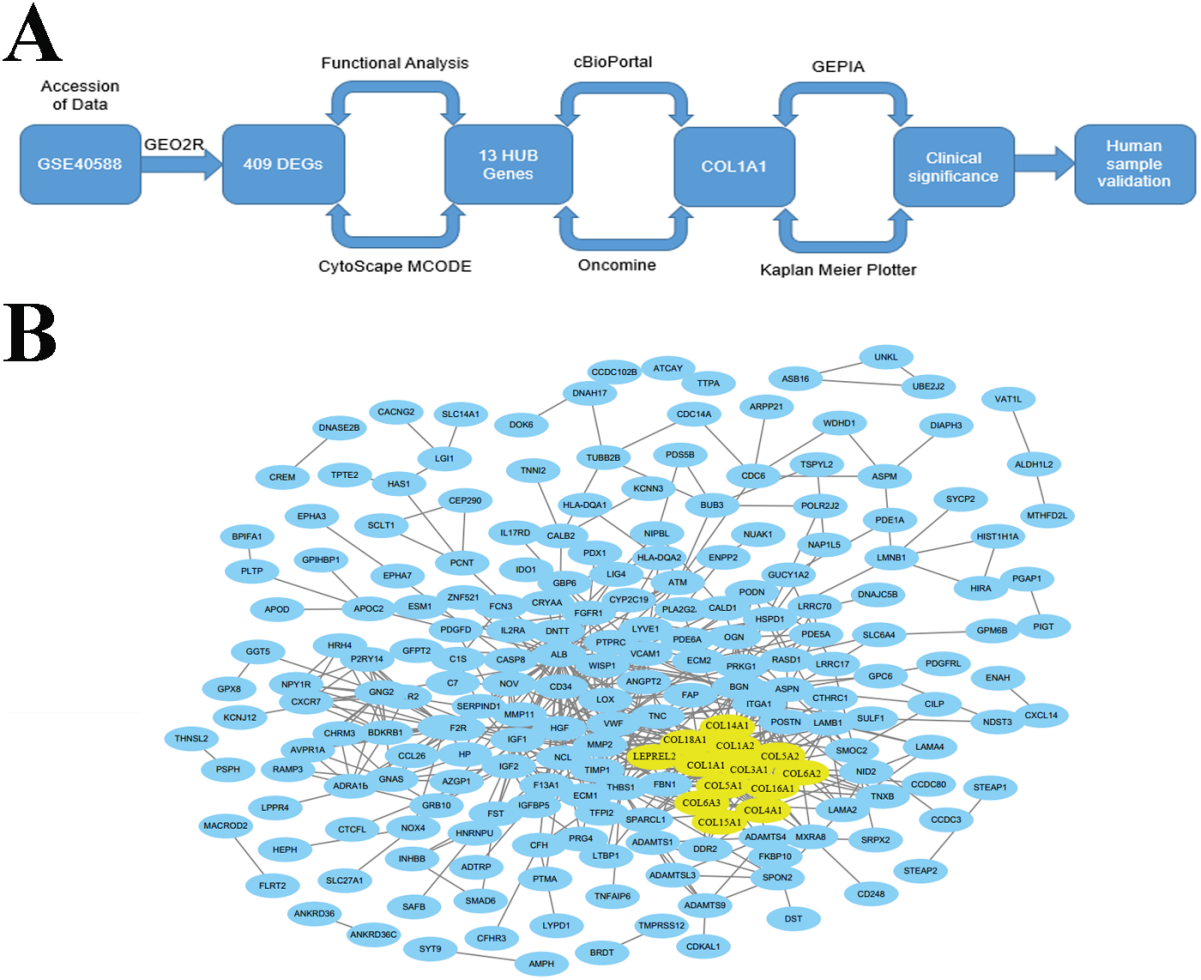
Identification of LSCC HUB Genes. (A) A methodical and strategic approach was developed to identify and validate genes of interest. 409 differentially expressed genes were found in GSE40588 that map to LSCC. (B) Of these 409 DEGs, 13 were identified as HUB genes (i.e., more than 13 unique connections).

The goal of the current work is to identify key biomarkers within the LSCC tumor microenvironment that may be used to help diagnose lymph node metastasis. We did this through an integrated approach (Fig. 1A) which used bioinformatic methods to analyze genome expression data and find genes which were differentially expressed and highly connected in late stage (i.e., stages II–IV) tumors. Through this work, we identified COL1A1 as a key biomarker within the LSCC tumor microenvironment. We further validate COL1A1 using clinical biopsy samples and demonstrate that COL1A1 expression correlates with lymphatic metastasis and poor clinical outcomes. It is our hope that this research can be used to supplement current LSCC assessments and provide both prognostic and diagnostic value.

## Materials & Methods

### Microarray data

GSE40588 was accessed and downloaded from the Gene Expression Omnibus database (https://www.ncbi.nlm.nih.gov/geo). The dataset contains 60 tumor-adjacent (histologically normal) noncancerous biopsy samples. 34 (56.7%) were from patients diagnosed with regional lymph node metastasis. 57 of the cases were male vs 3 female with an average age of 58.9 years. 16 (27%) of the cases were designated N1, 18 (30%) were designated N2, and 26 (43%) were designated N0. All samples were analyzed using an Agilent 4x44K gene expression microarray. Primary data are available in the supporting information.

### Identification of DEG and HUB genes

Differentially expressed genes (DEGs) were identified using GEO2R (Barrett et al., 2013). Cutoff criteria was set to a *P*-value <0.05 and logFC (fold change) >0.5. Probe sets which were misidentified were excluded (i.e., gene symbols not in the Genome Database) and genes with two or more probe sets were averaged. The Search Tool for the Retrieval of Interacting Genes was used to construct an interaction network from the DEG’s (Szklarczyk et al., 2015; Doncheva et al., 2019). Cytoscape version 3.4.0 software was used to visualize the output sequence. Molecular Complex Detection (MCODE Bader & Hogue, 2003) was used to arrange the topology, cluster the connected genes, and search through the resulting network. Proteins were included if they had an MCODE score >5, degree cutoff of 2, node score cutoff of 0.2, node density cutoff of 0.1, k-score of 2 and max depth of 100. HUB genes were initially selected if their degree was >10. Functional analysis was performed on both the DEG and HUB genes using the Database for Annotation, Integrated Discovery, and Visualization (DAVID; http://david.ncifcrf.gov) and validated using Cytoscape’s Biological Networks Gene Oncology (BiNGO) tool (Maere, Heymans & Kuiper, 2005). HUB gene and Cytoscape data are available in the supporting information.

### Identification of COL1A1 in LSCC

HUB genes were selected which have a degree of connectivity of ≥10. cBioPortal was used to screen the cancer genome atlas pan cancer atlas (Rhodes et al., 2004). This contains 487 unique lung squamous cell carcinoma samples. Following identification of COL1A1 in LSCC, OncoMine was used to evaluate overall expression levels in the sample set. Additionally, UCSC Xena (Goldman et al., 2020) was used to evaluate isoform expression levels. Furthermore, a receiver operating characteristic (ROC) curve was generated by ploting the sensitivity vs specificity of COL1A1 using The Cancer Genome Atlas (TCGA) database.

### Immunostaining of clinical samples

Immunostaining of clinical biopsy samples was done using general protocols. Briefly, tissue samples were fixed using a 4% paraformaldehyde solution. Tissues were frozen and 5 µm sections were cut using a cryostat. Sections were placed on a histological slide and dried for 30 min on a slide warmer. Slides were then immediately stained to reduce chances of oxidation and epitope loss. Sections were blocked using a blocking buffer (Fisher Scientific) and then stained with a mouse anti-COL1A1 (Proteintech, China) followed by an anti-mouse secondary antibody (IHC Kit, Maixin Company, China). UltraSensitive SP was used as a detection agent following the manufacturer’s recommended protocols.

### Validation in clinical samples

33 unique LSCC tissue biopsies were collected from patients with a median age of 59.09 (±7.30) undergoing lobectomy at the First Hospital of China Medical University. Collections took place between January 2012 and December 2012. The general information of clinicopathological of the patient are presented in Table 1. COL1A1 expression was detected using the aforementioned immunostaining protocol, and levels were evaluated using a general H-score protocol. Briefly, 5 regions of each sample were randomly chosen and visualized at 400x magnification. Intensities were based on a range of 0–3 where 0 is no staining, 1 is weak staining, 2 is moderate staining, and 3 is strong staining. Staining ratios were based on ranges from 0–100. H-score was calculated as the product of intensity and staining ratio (i.e., % of cells stained positive in a given region of interest). The research was approved by the Ethics Committee of the First Hospital of the China Medical University (AF-SOP-07-1. 1-01). Written informed consent was obtained from all of the patients. Raw data for human samples are available as a spreadsheet in the Supplemental Information.

**Table 1:** The general clinicopathological information of the patients.

	N0	N1	N2
Cases	16	11	6
Ages	56.7 ± 9.7	57.8 ± 5.9	60.8 ± 7.2
Sex (male: female)	15:1	11:0	6:0
Smoker: Non-smoker	14:2	10:1	6:0
Grade (Well-Moderate: Poor)	9:7	6: 5	4:2
Tumor size (<3 cm: ≥3 cm)	3:13	2:9	1:5

### Statistical analysis

Statistical analysis of *in vitro* work was performed using SPSS (Version 20). Unless otherwise noted, all experiments were independently replicated at least 5 times (*n* = 5). Data is plotted as the mean and SEM. Results were deemed statistically significant if *p* < 0.05 using either a Mann–Whitney test. Statistical analysis of bioinformatic work was performed and reported by the integrated bioinformatic tool. All statistical measures are noted either within the figures or within the accompanying captions.

## Results

### Differential expression of collagen genes is associated with SCC

Accession of genome wide expression data is a powerful tool for evaluating biomarkers in a variety of different diseases. We analyzed GSE40588 (Cao et al., 2017), which profiles tumor-adjacent normal lung tissue from patients with squamous cell lung cancers, using GEO2R. 409 of the genes studied were differentially expressed in late stage LSCC, and 13 of these genes were highly connected HUB genes with >10 connections (Fig. 1B). Out of the identified HUB genes, COL1A1 was the most highly connected, with 31 degrees of unique connections.

**Figure 2:**
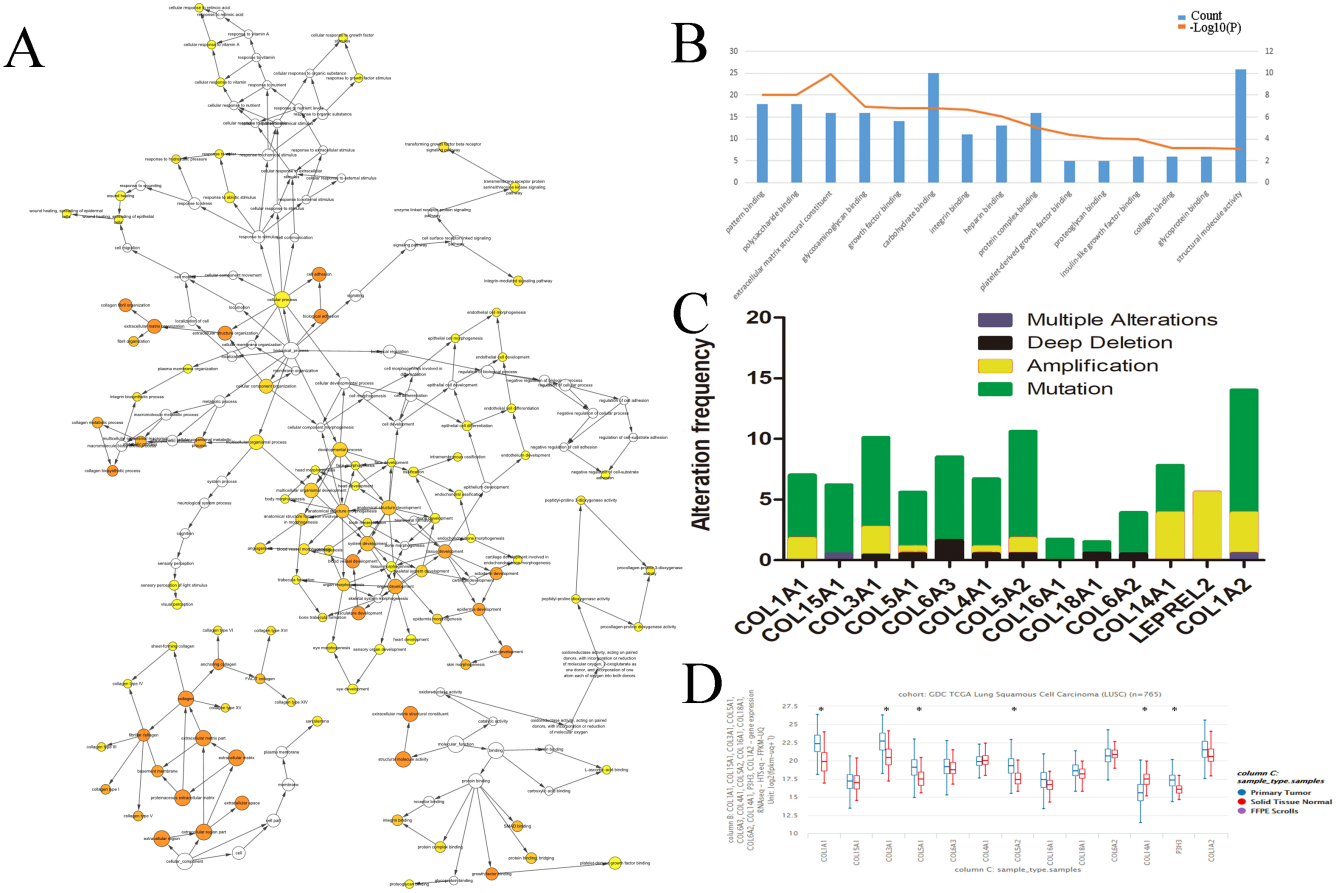
Identification of COL1A1 as a key marker within the LSCC tumor microenvironment. Relevant HUB genes were evaluated for their gene ontology using Cytoscapes BiNGO plugin (A) and DAVID (B). These resulted in an over representation of extracellular matrix, adhesion, and structural activities. (C) cBioPortal identified over 50 different alterations within the identified HUB genes, with mutations being the most prevalent. (D) However, many of these genes only had marginally higher expression levels in primary tumor vs. solid normal tissue. COL1A1 was chosen as the primary focus due to its high number of pathway connections and its high expression in the tumor microenvironment.

We evaluated the HUB genes using Cytoscape’s Biological Networks Gene Ontology (BiNGO). BiNGO assess over-represented or under-represented gene ontology categories and creates an interactive visual map for further evaluation. Over-represented ontologies are presented as darker color labels (i.e., orange) and less represented ontologies are lighter (i.e., yellow). While several of the gene functions were related, the most overrepresented were in extracellular matrix, adhesion, and structural activities (Fig. 2A). These results were expected considering the biological function of the collagen markers analyzed. These results were corroborated using DAVID, which showed a high number of carbohydrate binding and structural molecule activity (Fig. 2B). Further evaluation using cBioPortal showed that each HUB gene studied had a high frequency of alteration (Fig. 2C). While these results suggest that mutations in these genes, particularly overexpression, could be a prognostic marker, analysis of the hub genes on UCSC’s Xena Browser shows many of the genes are also highly expressed in solid normal tissue (Fig. 2D). Several identified genes (e.g., COL4A1, COL18A1, and COL1A2 to name a few) are only marginally overexpressed in primary tumors compared to normal solid tissue. COL1A1 and COL3A1 were the most over-expressed. Based on this data, as well as the high degree of connectivity (Fig. 1B), we chose COL1A1 as the most pertinent biomarker in the LSCC microenvironment.

**Figure 3:**
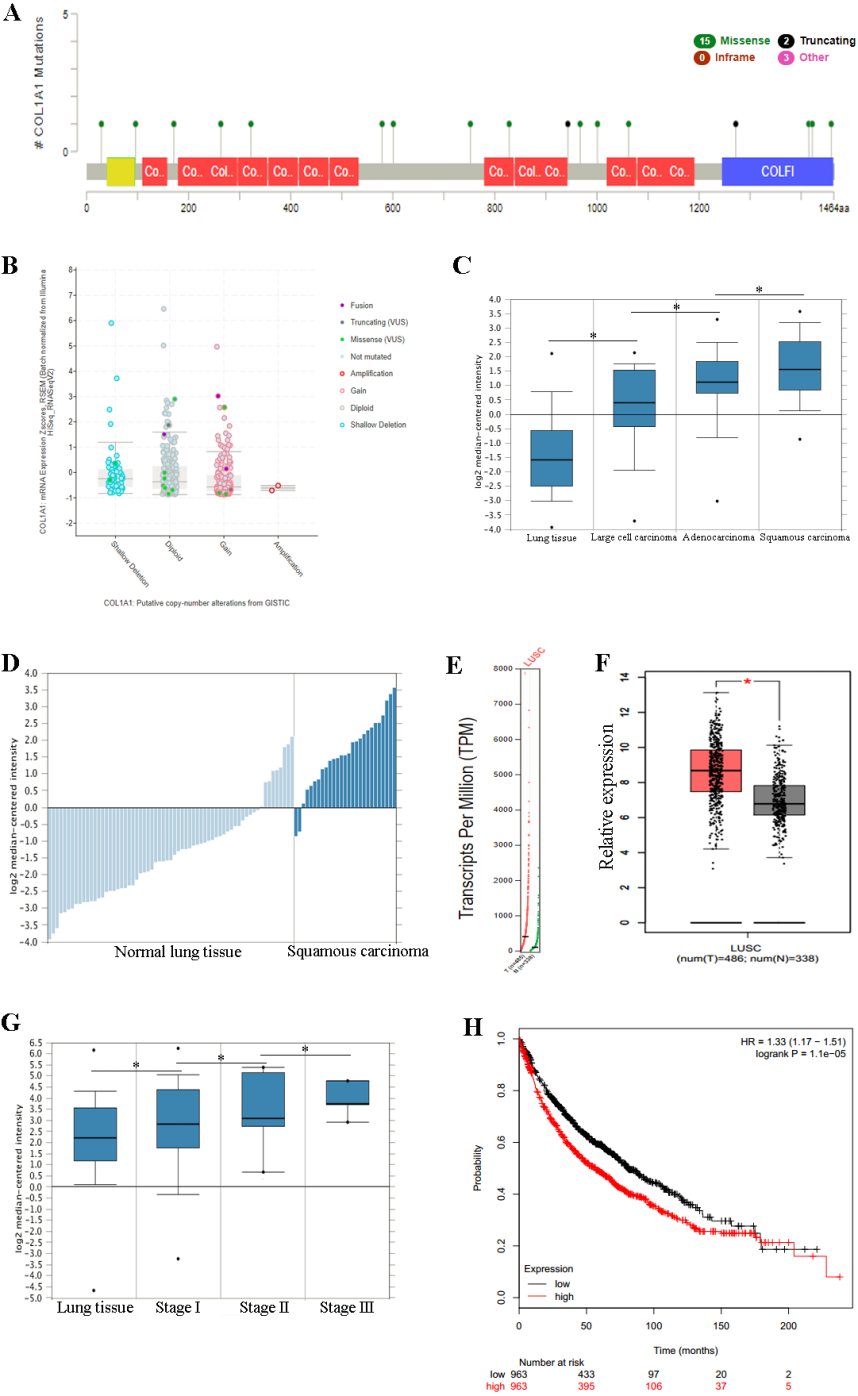
COL1A1 is associated with LSCC. Previous experiments have identified the over expression of COL1A1 as a diagnostic marker of LSCC. (A) There are 20 reported mutations with COL1A1, the most prevalent being missense mutations. (B) These mutations may result in a variety of genetic alterations, however most commonly the cells remain diploid without a homozygous or heterozygous deletion. (C) The overexpression of COL1A1 in the microenvironment is highest in LSCC, however it is also present in adenocarcinoma and large cell carcinomas. (D) LSCC samples have the highest median expression levels of COL1A1, nearly 3.5-fold higher than normal lung tissue. (E&F) This is the result of over transcription, where LSCC have much higher mean COL1A1 transcript levels. (G) Ultimately, data from the TCGA suggests that all LSCC, regardless of stage, demonstrate upregulation of COL1A1 in the tumor microenvironment. (H) While statistically significant, expression levels are nearly negligible in determining long term life expectancy (HR = 1.33).

### Overexpression of COL1A1 in the tumor microenvironment is a diagnostic marker for LSCC

We chose to further study COL1A1 as a prognostic and diagnostic marker for SCC. An examination of this gene using the UCSC Xena Browser shows that there are 20 unique COL1A1 mutations in the TCGA, with a missense mutation being the most common (Fig. 3A). In addition, we found that a majority of the genetic alterations correspond with a diploid presentation (Fig. 3B). While mutations in the COL1A1 gene may lead to other non-small cell lung cancers (e.g., large cell carcinomas and adenocarcinomas), it was nearly 2x as likely to mutated in squamous cell carcinomas (Fig. 3C). Furthermore, based on the Oncomine analysis, mean-expression levels are nearly 3.5-fold more in LSCC samples than in normal lung tissue (Fig. 3D, Corresponding *p*-values are provided in the figures). Comparatively, COL1A1 expression levels were higher in LSCC samples than in normal tissue samples, with a *p* value of 0.05. This conclusion was corroborated with GEPIA(Tang et al., 2017), which analyzes the TCGA gene expression levels. (Figs. 3E and 3F). Finally, we used computational tools to evaluate the prognostic value of COL1A1. Based on data from UCSC, we found a statistically significant difference (*p* < 0.05) between cancer and normal tissue. However, there was not enough difference to be able to discriminate between early and late stages of the disease. In addition, high expression of the COL1A1 gene only resulted in a marginal decline in mortality (HR = 1.33). These results suggest that COL1A1 expression may be a significant marker for identifying and validating NSCLC subtypes.

### COL1A1 upregulation signals metastasis to lymph nodes

**Figure 4:**
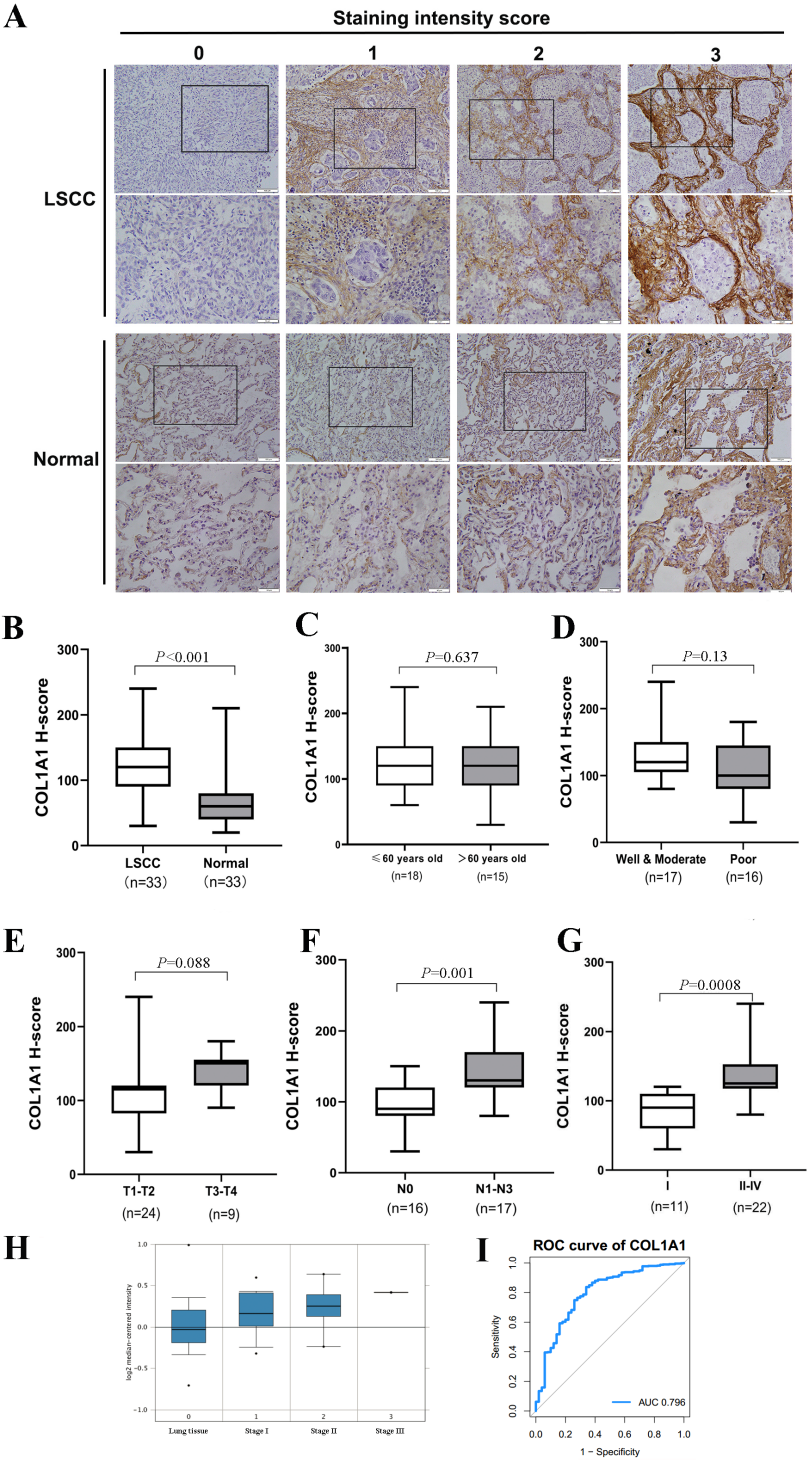
Clinical Validation of COL1A1. (A) COL1A1 was validated by grading 33 unique clinical biopsy specimens. The extent of reactivity was established through a general histo score (H-score) protocol (see Methods for details). This approach was used to determine the correlation between COL1A1 expression and (B) tumor presence (C) patient age (D) prognosis (E) tumor size (F) lymph metastasis, and (G) stage. Of these, COL1A1 expression was significantly higher in samples that had metastasized to at least 1 lymph node and in later stage tumors. (H) The results was further validated in Oncomine, with the increase of stage, the expression of COL1A1 was also up-regulated. (I) A ROC curve was produced with an area under the curve (AUC) value of 0.796.

Thus far, we have determined that COL1A1 is a potential diagnostic marker for NSCLC. This was determined using a number of applied bioinformatics strategies. There are several examples of similar workflows being done throughout literature (Cui et al., 2018; Gao & Wang, 2018). However, we chose to further validate these results using clinical biopsy specimens taken from patients with LSCC as well as matching adjacent normal tissue. Results from COL1A1 immunostaining (Fig. 4A) align with our bioinformatic results, suggesting LSCC samples have a much higher level of COL1A1 than did normal tissue (Fig. 4B). While there was no significant difference due to age, treatment, or tumor size (Figs. 4C–4E) there were significant differences in lymphatic metastasis and staging. Tumors with 0 lymphatic metastasis (N0) had a median H-Score of 100, whereas tumors which had metastasized to lymph nodes had a median score of 130. Early tumors (Stage I) also had a significantly lower median H-score, 80, than later stage tumors (Stage II–IV), 140. Compared to the earlier examination of genome sets (Fig. 3G), our data suggests stages II–IV have remarkably much higher COL1A1 expression levels. To corroborate these results, we evaluated the Oncomine database which contained 156 LSCC samples (Fig. 4H). Similar to the data presented in Fig. 4G, the expression levels of COL1A1 increase as the disease progresses. To further verify these results, we examined data in the TCGA database. A ROC curve was produced (Fig. 4I), with an area under the curve (AUC) values of 0.796, indicating high classification performance. These results suggest COL1A1 is a valuable marker which can be used to assist in diagnosing metastasis of LSCC tumors to the lymph nodes.

## Discussion

NSCLC’s such as LSCC are one of the most common causes of cancer-related mortality worldwide due to asymptomatic growth which leads to late-stage diagnosis (Klebe & Henderson, 2013). Advancements in screening technologies along with prophylactic screens of high-risk populations have significantly improved outcomes through identification of pre-invasive tumors (Barletta et al., 2017). In addition, the development of precision therapies and immunotherapies for lung adenocarcinomas have improved 5-year survival rates by targeting the root causes of the disease (Ruiz-Cordero & Devine, 2020). Despite these advances, only 25% of LSCC diagnoses are at an early-stage when the disease is localized (Blandin Knight et al., 2017), and treatments lag far behind lung adenocarcinomas (Leukam & Villaflor, 2015) demonstrating a collective lack of knowledge surrounding the disease.

First-line treatment of LSCC depends on the stage, location, and genome of the tumor. Surgical resection is often used to remove early-stage tumors, with lymph node sampling being done simultaneously. Staging of lymph node metastasis enables clinicians to optimize treatment protocols and provide patients with an accurate prognosis. However, micrometastases within the lymph node can be overlooked (Tufman & Huber, 2010), meaning that a percentage of patients who are identified as Stage 1 should be Stage II. Furthermore, tumors which advance to the lymph nodes are at risk for distal metastasis which significantly decreases 5-year survival rates. The use of diagnostic and prognostic markers may help rectify this error through accurate staging, ultimately leading to improved treatment regiments.

Genome wide screens have provided researchers with resources to rapidly identify disease markers and pathways inside the cell, which in turn have led to the discovery of potential oncogenes and markers for targeted therapies. However, to our knowledge, there have been no studies which investigate the LSCC tumor microenvironment in regards to lymph node metastasis. The tumor microenvironment provides a niche in which inflammatory cells, cytokines, and growth factors actively support rampant proliferation of tumors (Emon et al., 2018). In addition, the microenvironment provides cues which drive metastatic potential, extravasation, intravasation, and may even play a role in influencing therapeutic efficacies (Hirata & Sahai, 2017). Using GEO2R, we explored the gene expression profile of 60 LSCC patients and identified over 400 differentially expressed genes linked to extracellular proteins that were prevalent in late stages of the disease. Within this cluster of genes, we further identified 13 which we deemed highly connected HUB genes. The selected HUB genes were all within the collagen family of proteins aside from LEPREL2 (also known as P3H3). While not directly in the COL supergroup, LEPREL2 is involved in collagen biosynthesis, and specifically interacts with COL4A1 and COL1A1 (Tiainen et al., 2008). Therefore, it’s inclusion as a HUB gene rationally fits.

The goal of this work was to identify and evaluate a novel biomarker associated with lymph node metastasis. Using genome arrays, we ultimately identified COL1A1 as a relevant marker due to high expression in tumor samples and interconnectedness with other hub genes. This gene encodes the pro-alpha1 chains of type I collagen (Zhang et al., 2018b; Zhang et al., 2018a, p. 1). COL1A1 is often found in connective tissues (e.g., bone and tendon) and thus its mutation is most closely associated with osteogenic diseases, specifically osteogenesis imperfecta (Palomo, Vilaça & Lazaretti-Castro, 2017). Dysregulation of COL1A1 has been identified in several different cancers including breast (Liu et al., 2018, p. 1) and gastric (Li, Ding & Li, 2016, p. 1). In addition, its presence correlates with hypoxia in NSCLC (Oleksiewicz et al., 2017). Other authors have also suggested a tandem effect with COL1A2 in NSCLC and esophageal squamous cell carcinomas (Fang et al., 2019). In the present study, we found that not only does the expression of COL1A1 increase in NSCLC, but the presence is higher in LSCC than in other forms (Fig. 3C). In addition, our study shows that increases in COL1A1 expression often occurred later in tumor progression (Fig. 4G) and is associated metastasis to the lymph nodes (Fig. 4F).

Collagen is a key part of the extracellular matrix, providing structural support to surrounding cells. Density of collagen is dependent on the type of collagen (i.e., I–V) that comprises the network. Since COL1A1 is a more fibrous protein (Kuczek et al., 2019), it is reasonable to assume that high expressions of it within the LSCC microenvironment increases density of the surrounding tissue. COL1A1 presence may in fact be due to ongoing fibroplasia in the near area. Tumor specific ECM is known to be more collagen-rich and stiffer than normal tissue, which leads to increases in cellular proliferation and also reductions in immunosurveillance (Kuczek et al., 2019). Studies investigating the mechanistic link between fibrosis and lymph node metastasis will help in further validating COL1A1’s role in cancer progression. The identification of COL1A1 as a key part of this process may lead to druggable targets (i.e., Halofuginone (Zhang et al., 2018a)) which can delay progression of LSCC to the lymph nodes. In addition, it is a valuable prognostic marker which may aid clinicians in accurately staging the disease.

## Conclusions

Localized non-metastatic LSCC are often very treatable. However, tumors which have metastasized to the lymph nodes require both a different treatment paradigm and prognostic outlook. Here, we provide evidence that supports COL1A1 as a diagnostic marker that indicates the presence of lymph node metastasis. Furthermore, these results suggest that specific inhibitors (e.g., Halofuginone) and gene silencing may be potential strategies for decreasing metastatic potential. Further experimental and clinical studies are required to develop the required toolkit for accurately diagnosing and staging LSCC.

##  Supplemental Information

10.7717/peerj.10089/supp-1 Supplemental Information 1 Raw data from human samples

10.7717/peerj.10089/supp-2 Supplemental Information 2 The process to identify the hub genes

10.7717/peerj.10089/supp-3 Supplemental Information 3 STRING-interactions

10.7717/peerj.10089/supp-4 Supplemental Information 4 Hub genes

## Additional Information and Declarations

### Data Availability

The following information was supplied regarding data availability: The raw measurements are available in the Supplementary Files.
